# *Bx-daf-22* Contributes to Mate Attraction in the Gonochoristic Nematode *Bursaphelenchus xylophilus*

**DOI:** 10.3390/ijms20174316

**Published:** 2019-09-03

**Authors:** Mengge Gao, Yongxia Li, Wei Zhang, Pengfei Wei, Xuan Wang, Yuqian Feng, Xingyao Zhang

**Affiliations:** 1 Laboratory of Forest Pathogen Integrated Biology, Research Institute of Forestry New Technology, Chinese Academy of Forestry, Beijing 100091, China; 2 Co-Innovation Center for Sustainable Forestry in Southern China, Nanjing Forestry University, Nanjing 210037, China

**Keywords:** sex attraction, *Bursaphelenchus xylophilus*, RNA interference, *daf-22*, ascarosides

## Abstract

Studying sex communication is necessary to develop new methods to control the population expansion of gonochoristic species *Bursaphelenchus xylophilus*, the pathogen of pine wilt disease (PWD). Small chemical signals called ascarosides have been reported to attract potential mates. However, they have not been studied in the sex attraction of *B. xylophilus*. Here, we confirmed the sex attraction of *B. xylophilus* using a chemotaxis assay. Then, we cloned the downstream ascaroside biosynthetic gene *Bx-daf-22* and explored its function in the sex attraction of *B. xylophilus* through bioinformatics analysis and RNA interference. The secretions of females and males were the sources of sex attraction in *B. xylophilus*, and the attractiveness of females to males was stronger than that of males to females. Compared with *daf-22* of *Caenorhabditis elegans*, *Bx-daf-22* underwent gene duplication events, resulting in *Bx-daf-22.1, Bx-daf-22.2*, and *Bx-daf-22.3*. RNA interference revealed that the attractiveness of female secretions to males increased after all three *Bx-daf-22* genes or *Bx-daf-22.3* had been interfered. However, the reciprocal experiments had no effect on the attractiveness of male secretions to females. Thus, *Bx-daf-22* genes, especially *Bx-daf-22.3*, may be crucial for the effectiveness of female sex attractants. Our studies provide fundamental information to help identify the specific components and signal pathways of sex attractants in *B. xylophilus*.

## 1. Introduction

Chemical communications using small molecules are crucial for sex inter-attractions among invertebrate and vertebrate taxa [1,2,3]. Ascarosides, a family of small molecule signals that are derived from 3.6-dideoxysugar ascarylose and have fatty acid side chains with different lengths, are the main components of sex pheromones that are secreted by free-living and parasitic nematodes to attract mates [4,5,6]. Ascarosides are divided into many types including short-chain (the number of carbon atoms ≤12) and medium/long-chain ascarosides [7,8]. Previous study has shown that secretions of *C. elegans* hermaphrodites have strong attractiveness to males and the effective components were identified as short-chain ascarosides [4]. The short-chain ascarosides are derived from long-chain ascaroside precursors through the peroxisomal β-oxidation of the lipid side chain, a four-step process that truncates fatty-acids by two carbons [7,8]. Mass spectrometry-based analyses of the excretomes of *C. elegans* revealed that the four steps are catalyzed by enzymes ACOX, MAOC-1, DHS-28, and DAF-22 [6,7]. *Daf-22* encodes a homolog of the human sterol carrier protein SCPX, which is responsible for the final step in the peroxisomal β-oxidation process, and is necessary for the production of biologically active short-chain ascarosides and sex attraction in *C. elegans* [4,9].

Nematodes have evolved several reproductive modes including the two most common types, gonochorism (male–female mating) and self-fertilizing hermaphroditism as well as heterogeny (alternating gonochorism and hermaphroditism) and parthenogenesis (lack of sperm in reproduction) [10]. Hermaphroditic nematodes, like *C. elegans*, usually propagate by fertilizing their eggs using their own sperm. Gonochoristic species such as *Bursaphelenchus xylophilus* are universal in nature and locating mates is critical for their reproduction [11,12]. Sex attraction has been reported in many nematode species, and ascarosides are active components of sex pheromones that play important roles in the mating selection process by regulating sexually-associated behaviors [4,13]. The gonochoristic sour paste nematode *Panagrellus redivivus* produces female and male sex-specific ascarosides to attract the opposite sex [13]. In *C. elegans*, males are attracted to some short-chain ascarosides secreted by hermaphrodites [4].

The pathogen of pine wilt disease, *B. xylophilus*, is an invasive gonochoristic species that causes serious forest disease worldwide, particularly in countries in Asia and Europe [14,15,16]. The high reproductive rate of *B. xylophilus* is considered to be the main reason for the rapid death of pine trees [14,17]. Sexual attraction between the female and male of *B. xylophilus* has been investigated. Kiyohara (1982) indicated the mutual attraction between females and males by using an attraction assay of living nematodes [18]. Shinya (2015) also demonstrated the attractiveness of female and male secretions to the opposite sex [19]. However, the role of ascarosides in the sex attraction of *B. xylophilus* has not been studied. As ascarosides are the main active components of nematode sex pheromones, we attempted to explore their roles by studying the function of the downstream ascaroside biosynthetic gene *Bx-daf-22* in the sex attraction of *B. xylophilus*.

In this study, we first confirmed the attractiveness of the secretions from females and males to the opposite sex in *B. xylophilus*. Then, we revealed rapid gene duplication events involving substantial domain switching in *daf-22* in the β-oxidation pathway of ascaroside biosynthesis through bioinformatics-based comparisons between *C. elegans* and *B. xylophilus*. Finally, we explored the function of *Bx-daf-22* in the sex attraction of *B. xylophilus* by comparing the attractiveness of female and male secretions to the opposite sex before and after gene interference. Our results revealed that the three *Bx-daf-22* genes, especially *Bx-daf-22.3*, played roles in the attractiveness of female secretions to males in *B. xylophilus*, suggesting the main components of female sex attractants may be medium/long-chain rather than short-chain ascarosides.

## 2. Results

### 2.1. Secretions of Females and Males Attract the Opposite Sex in B. xylophilus

**Figure 1:**
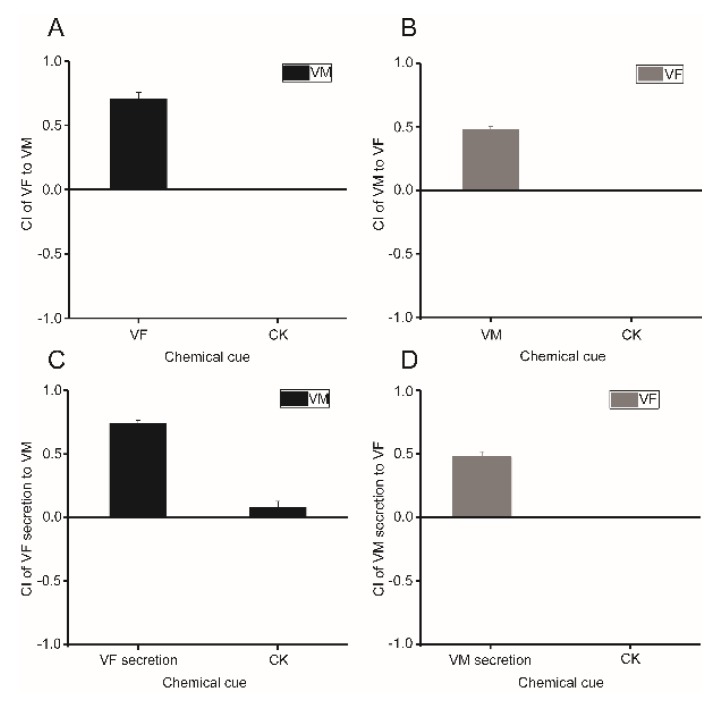
The attractiveness of VF, VM, and their secretions to the opposite sex. (**A**,**B**) The attractiveness of VF /VM to VM/VF. (**C**,**D**) The attractiveness of VF/VM secretion to VM/VF. CK: control of VF/VM: chemotaxis assay with M9 on the two spots. CK of VF/VM secretion: chemotaxis assay with ddH_2_O on the two spots. CI (chemotaxis index) = ((number of nematodes at the test zone) − (number of nematodes at the control zone))/total number of nematodes. VF: virgin female, VM: virgin male.

To identify the sex attraction between females and males of *B. xylophilus*, a two-spot assay was performed. Virgin females/males were assayed for their ability to attract the opposite sex. The results showed that the attractiveness of females to males was obvious (chemotaxis index (CI) = 0.704 ± 0.051) (Figure 1A), while the attractiveness of males to females was weaker than that of females to males (CI = 0.481 ± 0.023) (Figure 1B).

To further determine whether the attraction resulted from the secretions of the opposite sex, separate supernatant solutions of soaked females and males were independently used to assay the secretion activity. Results of the secretion attraction assay showed that the secretions of females and males attracted the opposite sex from a distance, and that the attractiveness of the female and male secretions were consistent with those of the female and male nematodes, with a CI value of 0.740 ± 0.024 (Figure 1C) and 0.475 ± 0.042 (Figure 1D), respectively.

### 2.2. Ce-daf-22 Duplication Events in B. xylophilus

**Figure 2:**
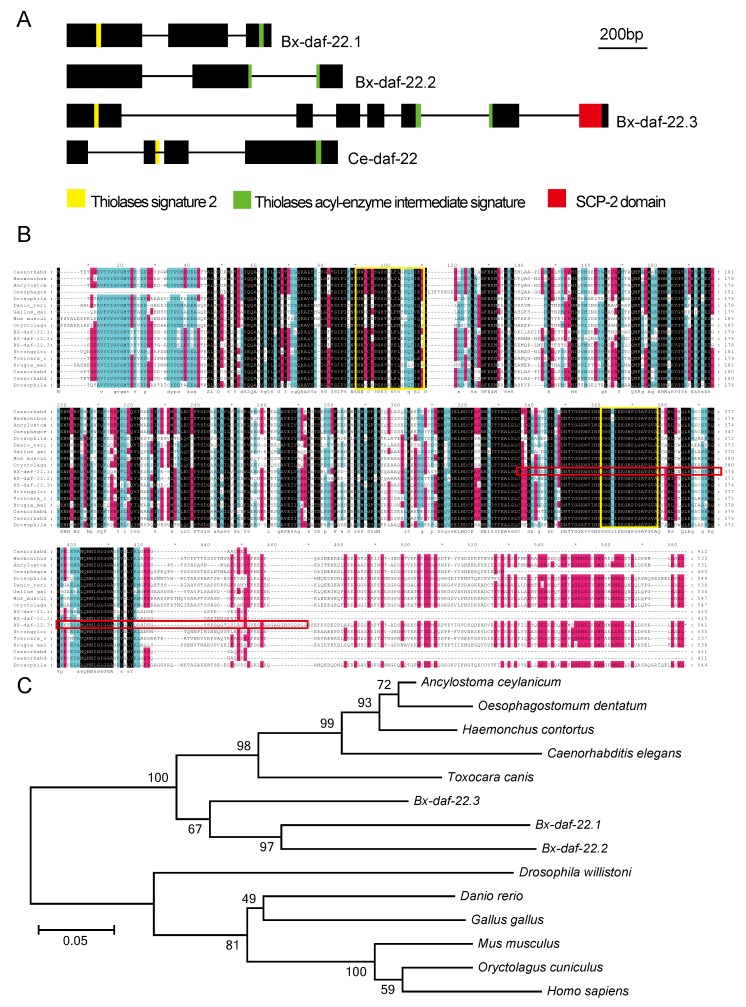
Characterization of *Bx-daf-22* genes in *B. xylophilus*. (**A**) The gene structure and protein domains of *Bx-daf-22.1*, *Bx-daf-22.2,* and *Bx-daf-22.3* in *B. xylophilus* compared with orthologs gene *Ce-daf-22* in *C. elegans*. Lines represent introns and black boxes represent exons. (**B**) Alignment of *B. xylophlus Bx-daf-22* with other species. Protein sequences were aligned using DNAMAN 8. Shaded parts indicate residues that are consistent. The first yellow box represents the thiolases acyl-enzyme intermediate signature and the second yellow box represents the thiolases signature 2. The red box represents the SCP2 domain. (**C**) Phylogenetic tree of *Bx-daf-22* and its homologues from other parasitic or free-living nematodes, insects and vertebrates. One thousand bootstrap replicates were performed, and the node labels represent the present bootstrap support. The accession number of these sequences are: (*Caenorhabditis elegans*, Maupas) NP_496639.1, (*Haemonchus contortus*, Rudolphi) AEO14647, (*Ancylostoma ceylanicum*, Looss) EYC23922.1, (*Oesophagostomum dentatum*, Goodey) KHJ80319.1, (*Drosophila willistoni*, Sturtevant) XP_002065586.1, (*Danio rerio*, Hamilton) NP_957159.1, (*Gallus gallus*, Linnaeus) NP_001292129.1, (*Mus musculus*, Linnaeus) NP_035457.1, (*Oryctolagus cuniculus*, Linnaeus) NP_001075504.1, (*Strongyloides ratti*, Grassi and Segre) CEF60652.1, (*Toxocara canis*, Werner) KHN79232.1, (*Brugia malayi*, Brug) XP_001902124.1, (*Caenorhabditis briggsae*, Dougherty and Nigon) XP_002631757, (*Caenorhabditis remanei*, Sudhaus) XP_003109848, and (*Drosophila mojavensis*, Patterson) XP_002003825.

To study the function of *Bx-daf-22*, a homologous phylogenetic relationship analysis of *daf-22* between *B. xylophilus* and *C. elegans* was first evaluated. *Ce-daf-22* presents in a single copy in the genome of *C. elegans*, whereas it underwent gene duplication and domain shuffling events in *B. xylophilus*. In *B. xylophilus*, *daf-22* duplication events resulted in three genes: *Bx-daf-22.1*, *Bx-daf-22.2*, and *Bx-daf-22.3*. The former two genes only contained two thiolase domains, whereas *Bx-daf-22.3* has additional SCP-X and SCP-2 domains (Figure 2A). Thus, the comparison indicated a genetic differentiation between *C. elegans* and *B. xylophilus*.

### 2.3. Characterization of Bx-daf-22 Genes in B. xylophilus

The full lengths (from ATG to TAG/TAA) of the three genes were 1500 bp for *Bx-daf-22.1*, 2252 bp for *Bx-daf-22.2*, which both contained three exons and two introns, and 5256 bp for *BX-daf-22.3*, which contained seven exons and six introns. The structure of *Bx-daf-22.3* including the full SCPX and SCP2 conserved domains, was more complicated than that of *Ce-daf-22* (four exons and three introns) (Figure 2A). The cDNA of *Bx-daf-22.1* was 1242 bp in length and encodes a protein of 413 amino acids, with a mass of 44.467 kDa, as predicted in the PROSITE database (https://prosite.expasy.org/). *Bx-daf-22.2* cDNA was 1248 bp in length and encodes a 415 amino acid protein with a mass of 44.403 kDa. *Bx-daf-22.3* was 1626 bp in length and encodes a 541 amino acid protein with a mass of 58.018 kDa. The alignment of the sequences of the three *Bx-daf-22* genes with sterol carrier proteins of other nematodes and organisms revealed that the three genes all included the thiolase acyl-enzyme intermediate signature and thiolase signature 2, but *Bx-daf-22.3* alone had a SCP2 domain (Figure 2B). Homologous analysis of the protein sequences indicated that *Bx-daf-22.1*, *Bx-daf-22.2*, and *Bx-daf-22.3* had significantly high similarity levels to *daf-22* sequences in other nematodes (Figure 2C) and their similarities to *C. elegans* were 68%, 68%, and 73%, respectively.

### 2.4. Expression Pattern of Bx-daf-22 through the Lifespan of B. Xylophilus

**Figure 3:**
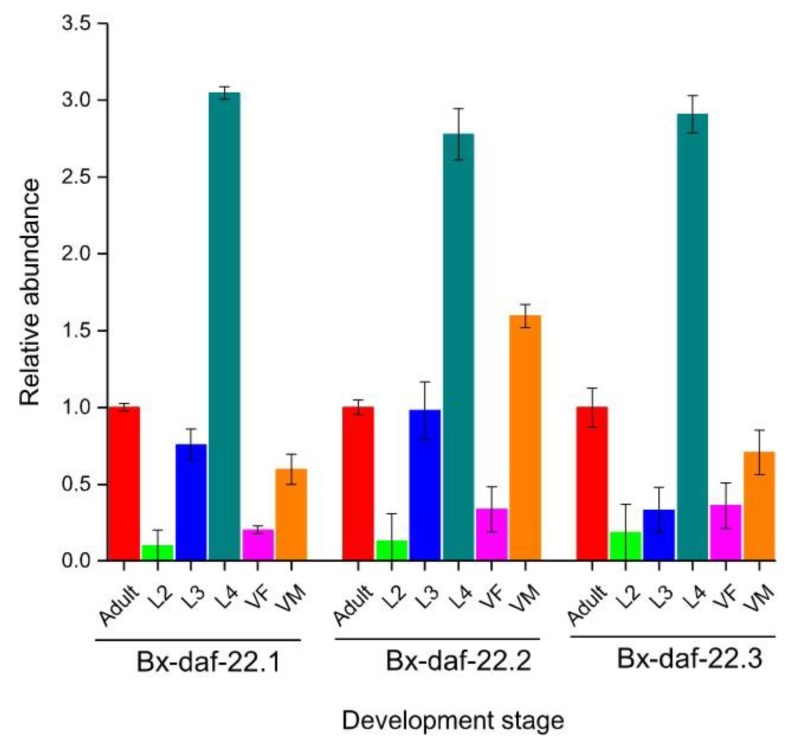
Transcriptional levels of *Bx-daf-22* genes at different developmental stages of *B. xylophilus*. The abundance of gene expression was quantified by quantitative real-time PCR (qRT-PCR) in different developmental stages and sexes of *B. xylophilus*: second-stage (L2), third-stage (L3), fourth-stage (L4), virgin female (VF), virgin male (VM), and adult. All gene expression levels were normalized to the β-actin gene.

The transcription levels of the three *Bx-daf-22* genes at different stages throughout the lifespan of *B. xylophilus* including the reproductive period of larval stages L2, L3, and L4, virgin male, virgin female, and mixed adult stages were assessed by qRT-PCR. The three genes were expressed throughout the lifespan of *B. xylophilus*, increased sharply at the L4 stage, and the expression level in the male was higher than that in the female (Figure 3).

### 2.5. The Effects of Bx-daf-22 Genes on the Attractiveness of Female and Male Secretions to the Opposite Sex

To further explore whether the sex attraction between males and females was associated with ascaroside biosynthesis, we studied the correlations between sex attraction and the expression of thiolase *Bx-daf-22* genes in *B. xylophilus*. 

**Figure 4:**
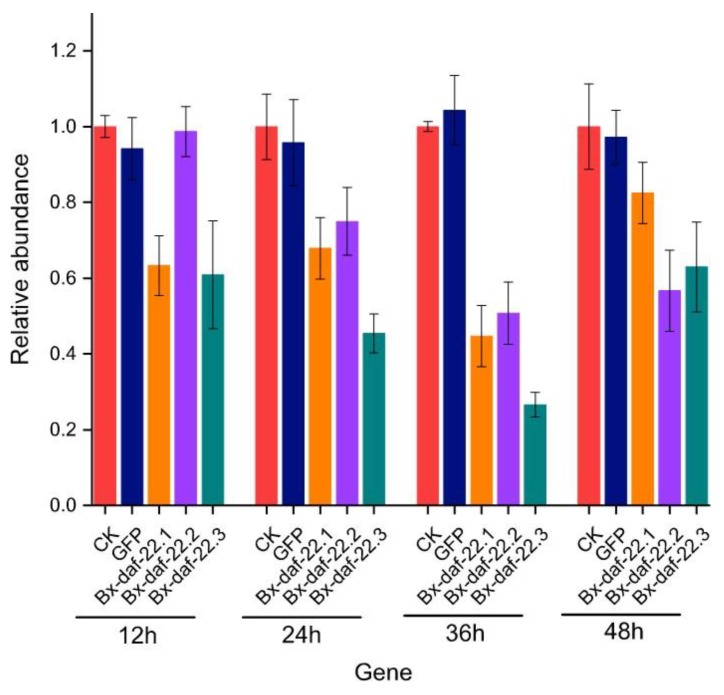
Expression levels of the *Bx-daf-22* genes at different times after soaking with the dsRNA of three genes simultaneously for 12, 24, 36, and 48 h. CK: the positive control, nematodes treated with M9 buffer alone; GFP: the negative control, nematodes treated with dsRNA *of gfp*; *Bx-daf-22.1*: nematodes treated with dsRNA of *Bx-daf-22.1*; *Bx-daf-22.2*: nematodes treated with dsRNA of *Bx-daf-22.2*; *Bx-daf-22.3*: nematodes treated with dsRNA of *Bx-daf-22.3*.

**Figure 5:**
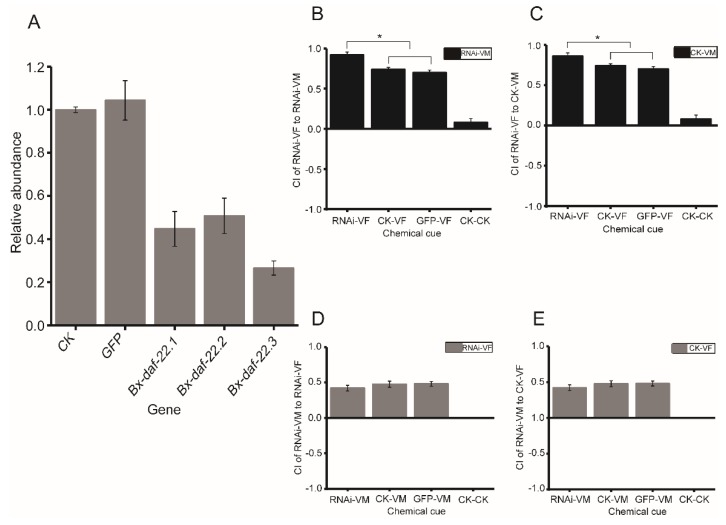
Expression levels of *Bx-daf-22* genes and effects of male and female secretions on the opposite sex after RNAi of all three *Bx-daf-22* genes. (**A**) Transcript abundance of *Bx-daf-22.1, Bx-daf-22.2*, and *Bx-daf-22.3* after the nematodes were treated with dsRNA of the three genes simultaneously for 36 h. (**B**,**C**) Attractiveness of secretions from RNAi-treated females to RNAi-treated and control males. (**D**,**E**) Attractiveness of secretions from RNAi-treated males to RNAi-treated and control females. CK: the positive control, nematodes treated with the M9 buffer alone. GFP: the negative control, nematodes treated with dsRNA of *gfp*. *Bx-daf-22.1, Bx-daf-22.2* and *Bx-daf-22.3*: The relative abundance of each gene after nematodes treated with dsRNA of *Bx-daf-22.1*, *Bx-daf-22.2,* and *Bx-daf-22.3* simultaneously. RNAi-VF/RNAi-VM: virgin female/male treated with dsRNA of three *Bx-daf-22* genes. CK-VF/CK-VM: virgin female/male treated with M9 alone. GFP-VF/VM: virgin female/male treated with dsRNA of *gfp*. CK–CK: the negative control, chemotaxis assay use ddH_2_O on the two spots. CI (chemotaxis index) = ((number of nematodes at the test zone) − (number of nematodes at the control zone))/total number of nematodes. VF: virgin female VM: virgin male. * *p*< 0.05.

As *Ce-daf-22* was replicated into three homeotic genes in *B. xylophilus*, we assumed that the function of *Ce-daf-22* was driven by the expression of the three *Bx-daf-22* genes. Therefore, nematodes were soaked in double-stranded (ds) RNA of *Bx-daf-22.1*, *Bx-daf-22.2*, and *Bx-daf-22.3* simultaneously. The interference efficiency levels against the three genes were first detected after soaking with their dsRNA for 12, 24, 36, and 48 h. The dsRNA of *Bx-daf-22.1* contained the thiolase acyl-enzyme intermediate signature, the dsRNA of *Bx-daf-22.2* contained the thiolase signature 2, and the dsRNA of *Bx-daf-22.3* contained the SCP2 domain. The expression levels of the three genes were lowest after 36 h of soaking (Figure 4). This indicated that the RNA interference (RNAi) by soaking was effective and specific for *B. xylophilus*. After the three genes underwent simultaneous interference, the gene expression levels of *Bx-daf-22.1*, *Bx-daf-22.2*, and *Bx-daf-22.3* were 44.72%, 50.76%, and 26.57%, respectively (Figure 5A). A chemotaxis assay with nematodes was then performed. When the three *Bx-daf-22* genes were subject to interference, the attractiveness of the secretions from RNAi-treated females to the control and RNAi-treated males increased obviously (CI = 0.92 ± 0.037 and CI = 0.86 ± 0.04, *p* < 0.05), compared with the attractiveness of secretions from the control females to the control males and RNAi-treated males (CI = 0.74 ± 0.075 and CI = 0.70 ± 0.024, respectively) (Figure 5B,C). However, the attractiveness of male secretions to female nematodes was not changed by the RNAi of *Bx-daf-22* genes (Figure 5D,E).

**Figure 6:**
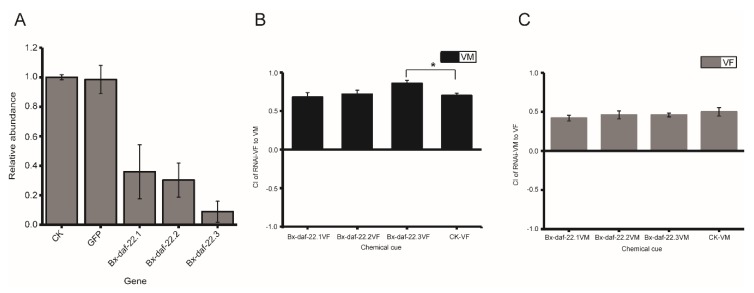
Expression levels of the *Bx-daf-22* genes and effects of male and female secretions on the opposite sex after RNAi of three *Bx-daf-22* genes individually. (**A**) Transcript abundance of *Bx-daf-22* genes after nematodes treated with dsRNA of individual gene for 36 h. (**B**) Attractiveness of secretions from RNAi-treated females on males. (**C**) Attractiveness of secretions from RNAi-treated males on females. CK: the positive control, nematodes treated with the M9 buffer alone. GFP: the negative control, nematodes treated with dsRNA *of gfp*. *Bx-daf-22.1*: nematodes treated with dsRNA of *Bx-daf-22.1*. *Bx-daf-22.2*: nematodes treated with dsRNA of *Bx-daf-22.2*. *Bx-daf-22.3*: nematodes treated with dsRNA of *Bx-daf-22.3*. *Bx-daf-22.1*-VF/VM: virgin female/male treated with dsRNA of *Bx-daf-22*.1. *Bx-daf-22.2*-VF/VM: virgin female/male treated with dsRNA of *Bx-daf-22*.2. *Bx-daf-22.3*-F/M: virgin female/male treated with dsRNA of *Bx-daf-22*.3. CK-VF/CK-VM: virgin female/male treated with M9 alone. CK-CK: the negative control, chemotaxis assay use ddH_2_O on the two spots. CI (chemotaxis index) = ((number of nematodes at the test zone) − (number of nematodes at the control zone))/total number of nematodes. VF: virgin female VM: virgin male. * *p*< 0.05.

Therefore, as the attractiveness of female secretions to males was influenced by *Bx-daf-22* genes, we further tried to determine which of the three genes played a major role in the attractiveness of female and male secretions in *B. xylophilus*. Nematodes were then soaked with the dsRNA of the individual *Bx-daf-22* genes, and the relative transcription levels of *Bx-daf-22.1*, *Bx-daf-22.2*, and *Bx-daf-22.3* after soaking for 36 h decreased to 35%, 30%, and 8% when compared with the control, respectively (Figure 6A). To investigate the effects of the three genes on the sex attraction between females and males, a chemotaxis assay using secretions from one sex of nematodes after RNAi to the opposite sex were performed. Results indicated the attractiveness of secretions from females to male nematodes increased significantly after the interference of *Bx-daf-22.3* (CI = 0.86 ± 0.049, *p* < 0.05), and no significant effects of the interference of the other two genes on the attractiveness of the secretions from females were found when compared with the control (Figure 6B). Additionally, the attractiveness of male secretions to females did not differ from the control (Figure 6C).

## 3. Discussion

Sex attraction is a key aspect in studying the sexual communication of gonochoristic species. Our study indicated that the secretions of females and males could attract the opposite sex in *B. xylophilus*, but the intensity that male nematodes responded to the secretions of females was much stronger than the response of females to the secretions of males. This might result from differences in the nervous system between females and males. Males have four additional sex-specific cephalic companion neurons in their heads, which have been hypothesized to mediate chemosensory mate finding behaviors in *C. elegans* [20,21,22]. In gonochoristic nematode species such as *P*. *redivivus*, females copulate a limited number of times during their lifecycle, whereas males often mate frequently. This indicates that male nematodes are always ready to copulate, showing a response to female attractants, whereas this is not the same for females [23,24]. In addition, the sex pheromones of females are usually effective, but male-produced pheromones appear to have no or only a weak ability to elicit a female mating-choice response [18,25,26,27]. Our previous research also suggested a female predominant mating system for *B. xylophilus* during its long term evolution [28].

Ascarosides are biosynthesized through peroxisomal β-oxidation, which is catalyzed by four distinct enzymes. In this study, we cloned the downstream enzyme *Bx-daf-22* (*Bx-daf-22.1, Bx-daf-22.2*, and *Bx-daf-22.3*) of the peroxisomal β-oxidation pathway and studied the function of *Bx-daf-22* in the sex attraction of *B. xylophilus*. Each of the three *Bx-daf-22* genes in *B. xylophilus* contain two thiolase domains and *Bx-daf-22.3* alone still has a C-terminal SCP-2 domain, which has been lost in in *C. elegans* and suggests the more advanced evolutionary position of *B. xylophilus* when compared with the free-living *C. elegans*. Furthermore, the expression levels of *Bx-daf-22* genes, especially *Bx-daf-22.3*, influenced the attractiveness of female secretions to males, suggesting that the main components of female attractants may also be ascarosides. Mutation of Ce-*daf-22* causes the absence of all short-chain ascarosides and increases the amount of ascarosides with long/medium chains [7]. As the attractiveness of females to males increased after the interference of *Bx-daf-22* in *B. xylophilus*, this demonstrates that long/medium-chain ascarosides may be the main components of female secretion to attract males.

In conclusion, we showed that the source of sex attraction were nematode secretions in *B. xylophilus*, identified as *Bx-daf-22*, which encodes the last step thiolase in the peroxisomal β-oxidation pathway of ascaroside biosynthesis, through bioinformatics comparisons, and confirmed the function of *Bx-daf-22* in the sex attraction of *B. xylophilus*. Females and males attract each other, but the attractiveness of females to males is stronger than that of males to females. Furthermore, the attractiveness of females to males was influenced by the expression level of *Bx-daf-22*, indicating that ascarosides may play important roles in the sex attraction of *B. xylophilus*. The medium/long-chain ascarosides may be the key components of female attractants. Additionally, *Bx-daf-22.3* may play a specific role in the biosynthesis of short-chain ascarosides related to the female attractants. These findings may provide a good base in which to further identify the specific components of attractants between the two sexes in *B. xylophilus*.

## 4. Materials and Methods

### 4.1. Nematode Source and Culture Conditions

The *B. xylophilus* strain NXY61 used in this study was isolated from a pine tree (*Pinus massoniana*, Lambert) in Ningbo, Zhejiang Province, China, and cultured on *Botrytis cinerea* using potato dextrose agar (PDA) Petri dishes at 25 °C in the dark for 5–7 d [29].

### 4.2. Nematode Collection at Different Developmental Stages

Nematodes were washed from the cultured Petri dishes into a new 9 cm glass Petri dish using 1× M9 buffer. After 20 min, the eggs were stuck onto the bottom of the glass Petri dish because of their glycoprotein surfaces. Then, the supernatant with nematodes of other stages was removed, and the dish was washed several times with 1× M9 buffer to obtain pure eggs. The eggs were soaked in 10 mL ddH_2_O and incubated at 25 °C in the dark for 24 h, and almost all eggs were hatched to L2 at that time. L2 were collected and inoculated onto PDA plates with *B. cinerea* for 24 h to obtain the L3, and for 52 h to obtain the later L4. As the later L4 can be distinguished as female and male by their genitalia, the same sexes were picked and cultured together for 24 h to obtain adult virgin female and male nematodes. These nematodes were used for next RNA extraction.

### 4.3. Attraction Assay

**Figure 7:**
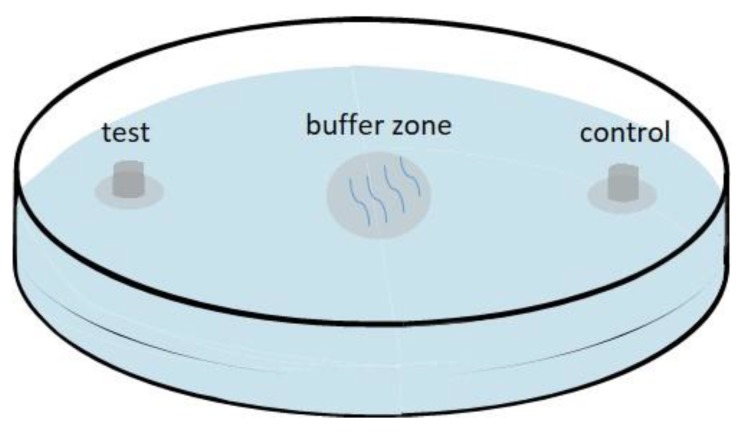
Schematic diagram of the attraction assay.

To test the attractiveness of virgin females and males, a two-spot assay was used as described previously [4,6,13]. Two glass cylindrical tubes (inner diameter 0.8 mm, outside diameter 1 mm) were placed on the opposite spots of a standard 60 mm Petri dish containing 2% agar, which was prepared the day before use to evaporate the water. The two opposite spots were apart from each other 3 cm from the center of the Petri dish. Then, 5 μL ddH_2_O containing five male/female nematodes was added to one of the glass tubes, and 5 μL ddH_2_O without nematodes was added to the other glass tube as the control. A drop of 0.8 μL 1 × tetramisole hydrochloride was added to both spots after 30 min to paralyze nematodes once they reached the test spots or control. Subsequently, 5 μL ddH_2_O with 10 nematodes of the opposite sex was put in the center of the plate (buffer zone) and 1.5 cm away from each spot in every trial. Then, the test plate was covered and stored at 25 °C in the dark for 20 min (Figure 7). The nematodes paralyzed within 1 cm of the test and control spots were scored. The chemotaxis index (CI) was defined as: ((number of nematodes in the test zone) − (number of nematodes in the control zone))/total number of nematodes. Females and males used in this study were harvested as L4 the day before use and cultured on the PDA petri dishes with *B. cinerea* at 25 °C in the dark to obtain young adults. Young adults were exposed to several washes and soaked in 1 × M9 buffer for 30 min to remove microbes. Each assay was repeated at least 10 times. To remove any bias, the control and test spots were interchanged after each trial. 

### 4.4. Secretion Attraction Assay

To test the attractiveness of the secretions from females and males, 100 virgin females and males were collected, and washed several times with 1× M9 buffer to remove bacteria, followed by three washes with ddH_2_O. The supernatant of the last wash after centrifuging was used as the control of the later attraction assay. Then, the nematodes were placed into 100 μL of ddH_2_O for 12 h at 25 °C. This supernatant solution was used for the secretion activity assays after centrifugation. The assays were performed as described above.

### 4.5. RNA Extraction and cDNA Synthesis

The nematodes of each developmental stage were frozen immediately with liquid nitrogen, and RNA was extracted using an RNeasy plus mini kit (QIAGEN, Hilden, Germany, NO. 74134) following the manufacturer’s protocol. RNA quality was determined with a NanoDrop ND 1000 spectrometer (Thermo fish, Massachusetts, MA, USA). The samples of high quality (OD 260/280 > 2 and OD 260/230 > 1.8) were used to synthesize cDNA. In brief, cDNA was synthesized from the extracted RNA according to the protocol of the PrimeScript™ RT reagent Kit with gDNA Eraser (TaKaRa, Kyoto, Japan, NO. RR047A). 

### 4.6. Gene Cloning and Phylogenetic Analysis

The amino acid sequence of *Ce-daf-22* was used as the query in a BLAST search against the whole genome of *B. xylophilus* to identify homologous genes in the Nation Center for Biotechnology Information database (https://blast.ncbi.nlm.nih.gov/Blast.cgi). The full-length coding sequences (CDSs) of the identified genes were obtained from WormBase (https://www.genedb.org/Homepage/Bxylophilus). Gene-specific primers (Table 1) were designed to amplify the full-length CDSs sequences using PCR of the cDNA of *B. xylophilus* with primeSTAR HS (TaKaRa, NO. R040A). The amino acid sequence of *Bx-daf-22* and other related species were aligned using Clustal-W. Phylogenetic analyses were carried out using the neighbor joining (NJ) method, based on the Jones–Taylor–Thornton (JTT) model in Molecular Evolutionary Genetic Analysis (MEGA 6).

### 4.7. Quantitative Real-Time PCR (qRT-PCR) of Nematodes at Different Development Stage

The qRT-PCR (25 μL) was performed to detect gene expression levels using the TB Green^TM^ Premix EX taq (TaKaRa, NO. RR420A) Light Cycler 480 System. The qRT-PCR three-step reaction procedure was performed under the following conditions: denaturing stage: 95 °C for 30 s, PCR stage: 40 cycles of 95 °C for 5 s and 60 °C for 30 s. Melting stage: 95 °C for 5 s, 60 °C for 60 s, and 95 °C for 0 s. Cooling stage: 50 °C for 30 s. Each sample was repeated with three technical repeats and three biological repeats. Bx-actin was used as the reference gene (Table 1). The mean threshold cycle (Cq) values of each development stage were used for further analyses.

### 4.8. dsRNA Preparation of Three Bx-daf-22 Genes

The dsRNA templates of the three *Bx-daf-22* genes were obtained based on the PCR amplification of the CDS products using specific T7 primers (Table 1). Mixed stages of the nematodes (the ratio of adults to juvenile was approximately 2:1) were used for RNAi [30]. The dsRNA products corresponding to the three genes were prepared using the T7 RiboMAX™ Express RNAi System (Promega, Madison, USA, NO.P1700), and stored at −80 °C.

### 4.9. Attraction Assay of Female and Male after Interfering of Bx-daf-22 Genes in B. xylophilus

The nematodes of the treatment groups were soaked in 1 × M9 buffer with dsRNA of *Bx-daf-22.1, Bx-daf-22.2*, and *Bx-daf-22.3* together or individually (2 mg/mL), while the nematodes of the control group were soaked in 1 × M9 buffer only. The number of the soaked nematodes was 15 individuals per microliter. After soaking for 36 h in an incubator at 25 °C at 150 rpm in the dark, nematodes were washed several times with sterile water to remove the external dsRNA. Approximately 2000 nematodes were used per RNA extraction, and the remainders were used for the next attraction assay.

After interfering with the three *Bx-daf-22* genes together or individually, the L4 females and males of RNAi and control group were picked and cultured for 24 h separately to obtain virgin females and males. The attraction assay after interfering with the three genes simultaneously included two parts: using the secretions of the females from the RNAi-treated or control group to attract RNAi-treated or control male nematodes, and using the secretions of males from the RNAi-treated or control group to attract RNAi-treated or control female nematodes. The attraction assay of the nematodes after interfering with the three genes individually also included two parts: using the secretions of female nematodes in the RNAi-treated group to attract the males in the control group, and using the secretions of males in the RNAi-treated group to attract females in the control group. The attraction assay was performed as described above.

### 4.10. Data Analysis

Statistical analyses of the transcription levels of the *Bx-daf-22* genes and parameters of *B. xylophilus* were carried out using one-way ANOVAs in SPSS 22.0. The threshold *p*-value < 0.05 was considered as statistically significant. Graphs were made using Origin 2017 and Adobe Illustrator Artwork 16.0.

## Figures and Tables

**Table 1:** Primers used in the experiments.

Primer Name	Sequences (5′–3′)
*Bx-daf-22.1F*	GTAATCGGAGTGGGTATGAC
*Bx-daf-22.1R*	GAAAGAGCAGCCCAAAGG
*Bx-daf-22.2F*	ATGTCCAAGCCAAAGGTC
*Bx-daf-22.2R*	GCAACAAGTCGTTTGTAG
*Bx-daf-22.3F*	ATGTCCAAGCCAAAAGTC
*Bx-daf-22.3R*	AGCTCAAAGCAAAGTTGTAA
*DS-daf-22.1-T7F*	TAATACGACTCACTATAGGGAATCCTGGTCAGCGAGAAC
*DS-daf-22.1R*	CATAGGTATTGTCTGCTCTGT
*DS-daf-22.1F*	ATCCTGGTCAGCGAGAAC
*DS-daf-22.1-T7R*	TAATACGACTCACTATAGGGACATAGGTATTGTCTGCTCTGT
*DS-daf-22.2-T7F*	TAATACGACTCACTATAGGGAGTGGTTGTGGTCAGTGAG
*DS-daf-22.2R*	ATCAGTTCTCCCGCCTTT
*DS-daf-22.2F*	GTGGTTGTGGTCAGTGAG
*DS-daf-22.2-T7R*	TAATACGACTCACTATAGGGAATCAGTTCTCCCGCCTTT
*DS-daf-22.3-T7F*	TAATACGACTCACTATAGGGATCTATCAACGAGCGGATG
*DS-daf-22.3R*	CAGGATGAAGTCGGAGTC
*DS-daf-22.3F*	TCTATCAACGAGCGGATG
*DS-daf-22.3-T7R*	TAATACGACTCACTATAGGGACAGGATGAAGTCGGAGTC
*DS-GFP-T7F*	TAATACGACTCACTATAGGGATGGTCCCAATTCTCGTGGAAC
*DS-GFP-R*	CTTGAAGTTGACCTTGATGCC
*DS-GFP-F*	TGGTCCCAATTCTCGTGGAAC
*DS-GFP-T7R*	TAATACGACTCACTATAGGGACTTGAAGTTGACCTTGATGCC
*Actin-QF*	TCCGTACCCTGAAGTTGGCTAACC
*Actin-QR*	AAGTGGAGACGAGGGAATGGAACC
*Bx-daf-22.1QF*	CCAATGCGTTGAACTCTG
*Bx-daf-22.1QR*	CCAATACCAATGTTGTGTTGAAGAG
*Bx-daf-22.2QF*	ACATCCTATTGGTGCTACTG
*Bx-daf-22.2QR*	CCAGGTTGTGCTGAAGTC
*Bx-daf-22.3QF*	ATGACTGCTTCGCTTCCA
*Bx-daf-22.3QR*	CGATTCCGAGGTTGTGTT
